# Vancomycin-associated nephrotoxicity in non-critically ill patients admitted in a Brazilian public hospital: A prospective cohort study

**DOI:** 10.1371/journal.pone.0222095

**Published:** 2019-09-05

**Authors:** Claudmeire Dias Carneiro de Almeida, Ana Cristina Simões e Silva, João Antonio de Queiroz Oliveira, Isabela Soares Fonseca Batista, Fernando Henrique Pereira, José Eduardo Gonçalves, Vandack Nobre, Maria Auxiliadora Parreiras Martins

**Affiliations:** 1 Faculdade de Medicina da Universidade Federal de Minas Gerais, Belo Horizonte, Minas Gerais, Brazil; 2 Hospital das Clínicas da Universidade Federal de Minas Gerais, Belo Horizonte, Minas Gerais, Brazil; 3 Núcleo Interdisciplinar de Investigação em Medicina Intensiva (NIIMI), Belo Horizonte, Minas Gerais, Brazil; 4 Faculdade de Farmácia da Universidade Federal de Minas Gerais, Belo Horizonte, Minas Gerais, Brazil; Azienda Ospedaliero Universitaria Careggi, ITALY

## Abstract

**Introduction:**

Vancomycin is widely used to treat infections caused by Gram positive bacteria, mostly methicillin-resistant strains. Despite its therapeutic effectiveness, vancomycin is a nephrotoxic drug that has been associated with the occurrence of acute kidney injury (AKI). In this study, we sought to evaluate the variability of serum trough concentrations of vancomycin and to determine the incidence and risk factors of vancomycin-associated nephrotoxicity (VAN) in non-critically ill patients.

**Methods:**

This was a prospective cohort including Brazilian public hospital inpatients from April 2017 to June 2018. The participants received intravenous vancomycin therapy for at least 48 hours for any suspected or confirmed infection by Gram positive bacteria. Demographic, clinical and laboratory data were collected. Information on vancomycin therapy and concomitant use of other nephrotoxic drugs were also recorded. Patients were followed up until discontinuation of vancomycin treatment or death, whatever occurred first. The primary outcome was the occurrence of AKI. We performed a Poisson regression to determine risk factors for AKI.

**Results:**

Overall, 98 participants were included in the study. Median age was 55.9 (interquartile range [IQR] 40.6–66.8) years and 58 (59.2%) were men. Most of them showed subtherapeutic (<10mg/L) or supratherapeutic (>20mg/L) trough levels of vancomycin; 42.9% and 15.3%, respectively. A total of 19 (19.4%) patients developed AKI. Poisson regression showed that male sex (odds ratio [OR] 2.90; confidence interval [CI] 95% 1.28–6.53; p = 0.011), concomitant use of piperacillin-tazobactam (OR 4.66; CI 95% 2.26–9.58; p <0.001) and vancomycin trough levels above 20mg/mL (OR 4.21; CI 95% 1.57–11.278; p = 0.004) were independently associated with AKI.

**Conclusions:**

Our study showed that usual doses of vancomycin did not reach recommended therapeutic serum trough levels of vancomycin in non-critically ill patients. Besides that, nephrotoxicity was common in this population, being associated with male sex, concomitant use of piperacillin-tazobactam and supra-therapeutic trough serum levels of vancomycin.

## Introduction

Vancomycin is a glycopeptide antibiotic widely used to treat infections caused by multidrug-resistant Gram positive microorganisms including methicillin-resistant *Staphylococcus* coagulase negative, *Enterococcus faecium* and, mainly, methicillin-resistant *Staphylococcus aureus* (MRSA). Most common infections involving these microorganisms include pneumonia, endocarditis, sepsis, osteomyelitis and meningitis [1]. Cross-sectional study developed in Brazil revealed that vancomycin is extensively used in tertiary hospitals, reaching 30.3% of intensive care unit (ICU) patients [2]. Despite its effectiveness, vancomycin has a significant risk of inducing nephrotoxicity [3]. The incidence of vancomycin-associated nephrotoxicity (VAN) varies from 5 to 43% in hospitalized patients, depending on clinical condition, particularities of setting and diagnosis criteria [4–6]. Doses >4g, serum trough concentrations >15mg/L and duration of therapy >7 days have been reported as risk factors for the occurrence of AKI in patients using vancomycin [7–9].

An international consensus guideline was published in 2009 to help achieving effectiveness and safety of vancomycin therapy [10]. This guideline recommended that trough serum concentrations should be considered as an accurate and practical method to be used in clinical practice. Trough concentrations should be obtained before the fourth or fifth dose, which corresponds to the *steady-state* concentration, in patients with normal renal function. Besides, trough serum concentrations should be maintained >10mg/L to avoid bacterial resistance and ideally targeted between 15 and 20mg/L for severe infections, such as bacteremia, endocarditis, osteomyelitis, meningitis and hospital-acquired pneumonia caused by MRSA [11]. Concentrations at this level would provide achieving area under the concentration curve (AUC) divided by minimum inhibitory concentration (MIC) [AUC/MIC] of ≥400 mgxh/L that is related with efficacy in most patients for MIC ≤1mg/L. However, in real world scenarios, it is challenging to achieve desirable vancomycin levels, which is confirmed by the majority of studies conducted on this issue [4,12,13].

Although several authors had investigated many aspects of vancomycin therapy, most data reported are from critically ill patients and based on retrospective studies [4,5,14]. Evaluation of VAN among population of non-critically ill patients obtained from prospective studies is lacking. Given that, the aim of this study was to evaluate the variability of serum trough concentration of vancomycin, to determine the incidence of VAN and to assess risk factors for this condition in non-critically ill patients.

## Methods

### Study design, setting and participants

We performed a single-center prospective cohort study to determine the incidence of VAN in non-critically ill patients hospitalized in a 509-bed tertiary hospital in Belo Horizonte, Southeastern Brazil. It is a referral public hospital, which provides clinical and surgical emergency assistance using evidence-based protocols to guide patient care. The hospital electronic database was used to identify potentially eligible patients. Accordingly, adults (age ≥18 years) who initiated intravenous vancomycin between April 2017 and June 2018 were assessed for potential eligibility. Inclusion criteria were: suspected or documented infection caused by Gram positive bacteria, receiving intravenous vancomycin therapy for at least 48 hours. Exclusion criteria were: patients who had estimated Glomerular Filtration Rate (eGFR) <30 mL/min/1.73m^2^, undergoing or not renal replacement therapy (RRT) prior to inclusion; patients undergoing continuous RRT or hemodialysis; patients admitted in critical care departments, including ICU, emergency room, stroke care and coronary care unit. This study protocol was approved by the Research Ethics Committee of the Universidade Federal de Minas Gerais (approval code CAAE 44346215.7.0000. 5149). All participants or their legal representatives signed an informed consent form to participate in the study.

### Data collection, variables and definitions

Demographic, clinical and laboratory data were collected from electronic medical charts records using a pre-tested questionnaire. Data were collected until three days after the end of treatment or death, whatever occurred first. Patients were characterized by sex, age and weight. Clinical data included: cause of admission, Charlson comorbidity index (CCI) [15] (obtained by the sum of the comorbidities identified according medical records), hemodialysis during vancomycin therapy. Laboratory data included: baseline values of serum urea, serum creatinine (SCr) and eGFR; vancomycin trough concentrations at the third (considered as initial trough level) and at the seventh day of treatment always until one hour before the administration of the dose of antibiotic. SCr, urea level and eGFR were also collected during the time of vancomycin use.

SCr was tested using CREA VITROS^®^ (Chemistry Products CREA Slides, Ortho-Clinical Diagnostics, Johnson & Johnson, Raritan, NJ, USA) according to the manufacturer’s recommendations. The estimated glomerular filtration rate (eGFR) was determined according to the CKD-EPI (Chronic Kidney Disease Epidemiology Collaboration) equation [16] using a calculator provided online by the Brazilian Society of Nephrology (https://sbn.org.br/utilidades/calculadoras/ accessed 2017–2018).

Vancomycin brand acquired by the study hospital was Novamicin^®^ (Novafarma Pharmaceutical Industry Ltda). Dosage regimen was determined by the assistant physician according to institutional protocols that recommends the use of 2g/day (1g every 12 hours) for most conditions. We assessed vancomycin trough levels measured at the third (after the fourth or fifth dose, corresponding to the *steady-state*) and at the seventh day of treatment. All blood samples were collected until one hour before the administration of vancomycin dose. Vancomycin levels were measured using VANC VITROS^®^ (Chemistry Products, Ortho-Clinical Diagnostics, Johnson & Johnson, Raritan, NJ, USA) according to the manufacturer’s recommendations.

Concomitant use of nephrotoxic drugs was assessed during vancomycin therapy. With the support of a nephrologist, we selected from the hospital formulary the drugs with a high potential to induce nephrotoxicity and frequent consumption in the hospital. The list included all formulations of amphotericin B represented by sodium deoxycholate amphotericin B, liposomal amphotericin B and amphotericin B lipid complex (S1 Table).

### Outcomes

The primary outcome was the incidence of AKI during vancomycin therapy or up to three days after this antibiotic interruption. AKI was defined as an absolute increase in SCr of ≥0.3mg/dL or ≥50% increase in serum creatinine from baseline in 48 hours [17]. Baseline SCr was recorded at initiation of vancomycin therapy. Secondary outcomes were length of stay and all causes of hospital mortality.

### Sample calculation

A sample size was calculated to estimate the number of eligible patients we should enroll during the study period. We took into account the AKI incidence of 0.12 observed in a pilot study with 34 patients admitted to the hospital in the same period of study. These patients were included in the final analysis of this study. Thus, with a power of 80%, significance level of 0.05 and precision of 0.10, a sample of at least 98 patients would be needed to confirm this incidence. We used the Z test for a ratio in the calculation. The software used was the Minitab Release 14 (Minitab LLC, State College, Pennsylvania, USA).

### Statistical analysis

We used descriptive methods to evaluate the variables. Continuous variables were tested for normality by Shapiro Wilk test and were presented as mean ± standard deviation (SD) or median (interquartile range—IQR), as appropriate. Subgroups were compared by Student’s *t* or the Mann-Whitney *U* test, when indicated. Categorical variables were presented as absolute frequency and percentage. We examined the association of categorical variables by Pearson’s Chi-squared test or Fisher’s exact test, when indicated. All variables associated with nephrotoxicity (p-value <0.20) in the univariate analysis were included in the multivariate analysis and were explored using Poisson regression. In the multivariate analysis, the backward elimination method was used to identify the model with best fit. In this method, one variable at a time was taken from the full model, starting with the one with the highest p-value. The final model was composed only of variables that presented a p-value <0.05. Goodness-of-fit was assessed by the Hosmer and Lemeshow statistic and the overall performance by Nagelkerke’s *R*^2^ Index. Non-significant Hosmer and Lemeshow statistic indicate a well-fitting model. Nagelkerke’s *R*^2^ values closer to 1 indicate better performance. All data were registered by double entry using EpiData software (ver. 3.1; EpiData Assoc., Odense M., Denmark). Statistical analyses were performed using IBM SPSS Statistics for Windows, Version 18.0 (IBM Corp, Armonk, NY).

## Results

**Fig 1:**
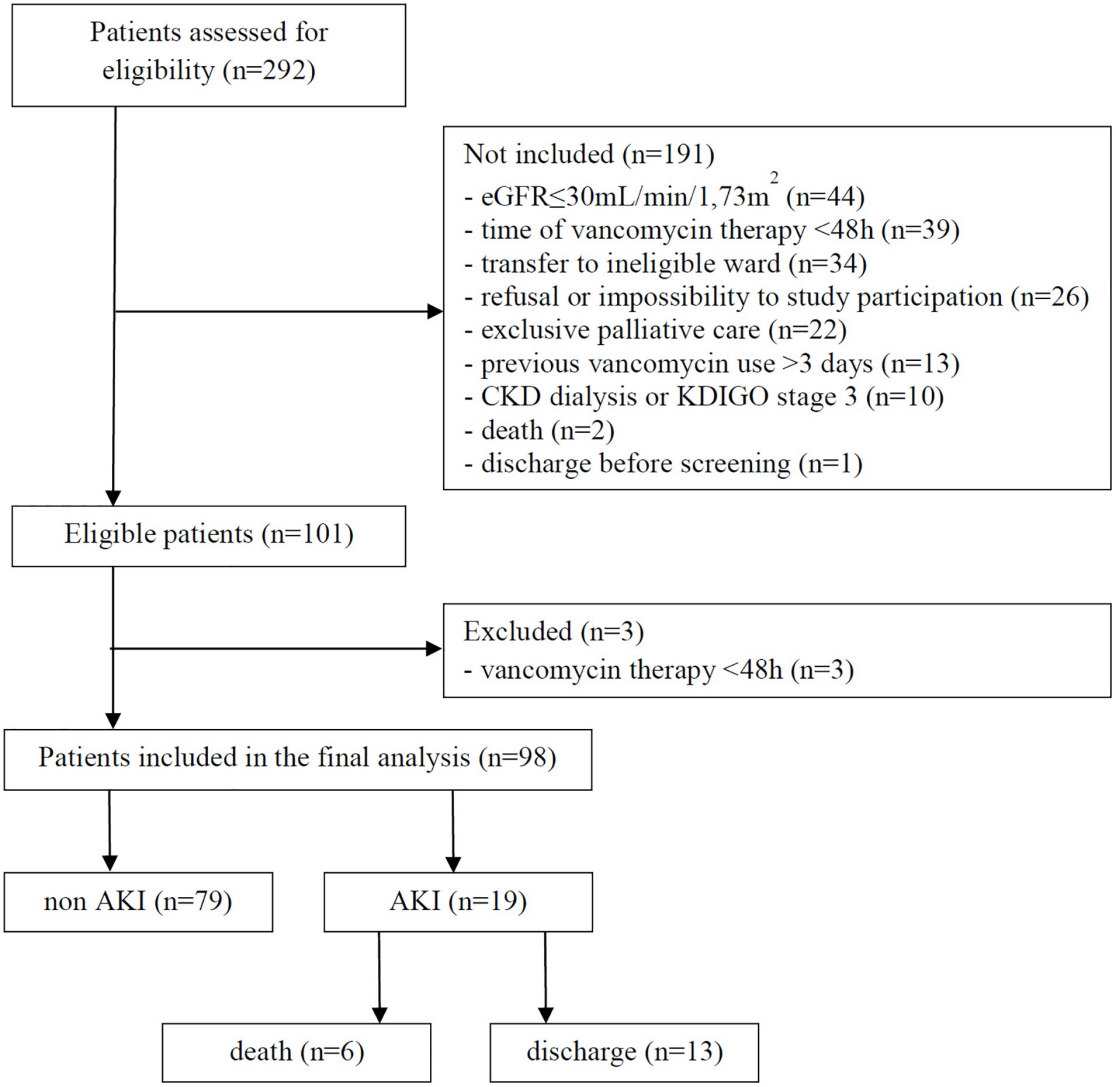
Flowchart for patient selection. eGFR, estimated glomerular filtration rate; KDIGO, Kidney Disease Improving Global Outcomes; CKD, chronic kidney disease; AKI, acute kidney injury.

During the study period, 292 patients admitted at the hospital wards were assessed for eligibility and 101 met the inclusion criteria. However, three patients were excluded from the final analysis due to a time of vancomycin use of less than 48 hours. Fig 1 depicts the flowchart for patient selection.

Studied patients were predominantly male (59.2%) with median age of 55.9 years old (interquartile range [IQR] 40.6–66.8), and most of them were medical admissions (68.4%). Median CCI was 2 (IQR 1–3). Most participants showed hospital-acquired infections (63.3%) and 58.2% had their infections microbiologically confirmed. The infection was caused by MRSA in 6.1% patients. According to evidence-based hospital protocol, vancomycin was initially prescribed as an empirical treatment due to the severity profile of patients and suspected infection by MRSA infection, which has not been confirmed in the microbiological results. In most of these cases, de-escalation was performed which is demonstrated by the average duration of vancomycin therapy <7 days. The most common sites of infection were skin and soft tissue (19.4%), surgical site (13.3%), pulmonary (12.2%), bone (7.1%) and catheter (7.1%). The main characteristics of the included patients are shown in Table 1.

**Table 1:** Descriptive data of study participants and comparison between AKI and non-AKI groups.

Characteristics	Total (n = 98)	AKI (n = 19)	Non-AKI (n = 79)	p-value
Age (years), median (IQR)	55.9 (40.6–66.8)	61.4 (55.3–71.3)	55.4 (38.9–66.0)	0.156^e^
Male sex, n (%)	58 (59.2)	15 (78.9)	43 (54.4)	0.051^f^
Weight (kg), median (IQR)	70 (60–80)	69 (60–80)	70 (60–80)	0.727^e^
Admission, n (%)				
Medical	67 (68.4)	13 (68.4)	54 (68.4)	0.996^f^
Surgical non-trauma	31 (31.6)	6 (31.6)	25 (31.6)	
Charlson com orbidity index	2 (1–3)	3 (2–5)	2 (0–3)	0.062^e^
Sepsis, n (%)	24 (24.5)	6 (31.6)	18 (22.8)	0.553^g^
Septic shock, n (%)	2 (8.3)	0 (0)	2 (2.5)	0.588^g^
Hospital-acquired infection, n (%)	62 (63.3)	15 (78.9)	47 (59.5)	0.114^f^
Microbiologically confirmed infection, n (%)	57 (58.2)	11 (57.9)	46 (58.2)	0.539^g^
MRSA confirmed infection, n (%)	6 (6.1)	2 (10.5)	4 (5.1)	0.329^g^
Positive blood culture, n (%)	21 (27.6)	6 (31.6)	15 (19.0)	0.230^g^
Infection site, n (%)				0.171^g^
Undetermined	21 (21.4)	2 (10.5)	19 (24.1)	
Skin/soft tissue	19 (19.4)	8 (42.1)	11 (13.9)	
Surgical site	13 (13.3)	3 (15.8)	10 (12.7)	
Pulmonary	12 (12.2)	1 (5.3)	11 (13.9)	
Bone	7 (7.1)	0 (0)	7 (8.9)	
Catheter	7 (7.1)	2 (10.5)	5 (6.3)	
CNS	6 (6.1)	1 (5.3)	5 (6.3)	
Kidney	5 (5.1)	0 (0)	5 (6.3)	
Others^a^	8 (8.2)	2 (10.5)	6 (7.6)	
Duration of vancomycin use (days), median (IQR)	6.0 (3.0–11.0)	8 (4–12)	6 (3–10)	0.186^e^
Concomitant antibiotics^b^				
Meropenem	55 (56.1)	10 (52.6)	45 (57.0)	0.933^f^
Cefepime	37 (37.8)	7 (36.8)	30 (38.0)	1.000^f^
Piperacillin-tazobactam	15 (15.3)	9 (47.4)	6 (8.9)	<0.001^g^
Polymyxin B	12 (12.5)	5 (26.3)	7 (10.1)	0.052^g^
Others^c^	12 (12.3)	1 (5.3)	11 (12.7)	0.451^g^
Number of potential nephrotoxic drugs	3 (2–4)	3 (2–5)	2 (2–3)	0.031^e^
Baseline urea (mg/dL)^h^, median (IQR)	37.0 (27.0–48,5)	41 (35–63)	34.5 (26–47.25)	0.083^e^
Baseline SCr (mg/dL)^i^, median (IQR)	0.70 (0.58–0.96)	0.74 (0.63–1.13)	0.70 (0.57–0.93)	0.069^e^
Baseline eGFR^d^ (mL/min/1,73m^2^), median (IQR)	118.20 (96.3–133.8)	108.5 (78.2–120.6)	122.1 (100.8–135.6)	0.028^e^
Baseline eGFR (mL/min/1,73m^2^)^I^, n (%)				
30.0–59.9	2 (2.1)	0 (0)	2 (2.6)	
60.0–90.0	16 (16.7)	6 (31.6)	10 (13.0)	
>90.0	78 (81.3)	13 (68.4)	65 (84.4)	
Initial trough vancomycin concentration (mg/L), n (%)	10.8 (7.65–14.58)	14.85 (11.21–21.97)	10.22 (7.19–13.46)	0.001^e^
<10	42 (42.9)	4 (21.1)	38 (48.1)	0.006^g^
10–14.9	33 (33.7)	6 (31.6)	27 (34.2)	
15.0–20.0	8 (8.2)	1 (5.3)	7 (8.9)	
>20.0	15 (15.3)	8 (42.1)	7 (8.9)	
Trough vancomycin concentration at day 7 (mg/L)^j^, median (IQR)	13.8 (9.3–19.9)	18.7 (7.2–30.2)	13.4 (9.5–16.8)	0.332^e^
Length of hospital stay, median (IQR)	32.5 (16.0–50.0)	35 (20–67)	32 (16–47)	0.316^e^
Renal replacement therapy, n (%)	1 (1.0)	1 (5.3)	0 (0)	0.194^g^
All cause mortality, n (%)	17 (17.3)	6 (31.6)	11 (13.9)	0.091^g^
AKI^d^, n (%)	19 (19.4)	-	-	-
stage 1	10 (52.6)	-	-	-
stage 2	3 (15.8)	-	-	-
stage 3	6 (31.6)	-	-	-

CNS, central nervous system; SCr, serum creatinine; eGFR, estimated glomerular filtration rate; AKI, acute kidney injury; IQR, interval interquartile

^a^Others infection sites included abdomen, ear and mouth.

^b^Total percentage is upper than 100% because a patient could use concomitantly more than one antibiotics.

^c^Others antibiotics included ceftazidime, ciprofloxacin, clarithromycin, metronidazole, polymyxin E, rifampicin and sulfamethoxazole-trimethoprim.

^d^KDIGO (Kidney Disease Improving Global Outcomes) criteria.

^e^Mann-Whitney’s U Test

^f^Chi-square Test

^g^Fischer Test

^h^Sample size of 93 patients

^i^Sample size of 96 patients

^j^Total of 57 patients were collected at seventh day of treatment

### Vancomycin use

Dosing of vancomycin regimen was predominantly 2g/day (1g every 12 hours) and only 13 patients used an alternative therapeutic schema including 1.0g (n = 6/98), 1.5g (n = 3/98) or 2.4g (n = 4/98) per day. Among patients receiving 2g/day of vancomycin, 37 (43.5%) attained target trough serum concentrations (10-20mg/L). Subgroup analysis according to target vancomycin trough serum concentrations and dosage could not be performed due to the small number of patients in each subgroup. The median vancomycin dose per weight was 28.57 mg/Kg (IQR 25.00–33.33) and there was no significant difference between groups with and without AKI (29.94 vs. 28.57, p = 0.993). No patient used loading doses and five were treated with monotherapy. Vancomycin trough serum concentrations varied considerably among study participants: 42.9% (42/98) have shown levels <10mg/L and 15.3% (15/98) ≥20mg/L. Median initial vancomycin trough serum concentrations were 10.8 mg/L (IQR 7.65–14.58).

Use of concomitant potentially nephrotoxic drugs was observed in 88 patients, and varied from one to six drugs (Table 2). The most common nephrotoxic drugs observed in this study were omeprazole (58.2%), cefepime (44.9%), furosemide (20.4%), losartan (15.3%), piperacillin-tazobactam (13.3%) and polymyxin B (13.3).

**Table 2:** Frequency of potentially nephrotoxic drugs used concomitantly with vancomycin in non-critically ill patients (n = 98).

Potentially nephrotoxic drugs	Frequency, n (%)
Omeprazole	57 (58.2)
Cefepime	44 (44.9)
Furosemide	20 (20.4)
Losartan	15 (15.3)
Piperacillin-tazobactam	13 (13.3)
Polymyxin B	13 (13.3)
Acyclovir	12 (12.2)
Allopurinol	10 (10.2)
Acetylsalicylic acid	9 (.2)
Sulfamethoxazole-thrimethoprim	8 (8.2)
Ketoprofen	6 (6.1)
Cyclosporine	6 (6.1)
Enalapril	5 (5.1)
Amphotericin B	4 (4.1)
Captopril	4 (4.1)
Ciprofloxacin	4 (4.1)
Amoxicillin- clavulanic acid	3 (3.1)
Ceftriaxone	3 (3.1)
Tacrolimus	3 (3.1)
Gancyclovir	1 (1.0)
Gentamicin	1 (1.0)
Polymyxin E	1 (1.0)
Rifampicin	1 (1.0)

### Vancomycin trough serum levels

**Fig 2:**
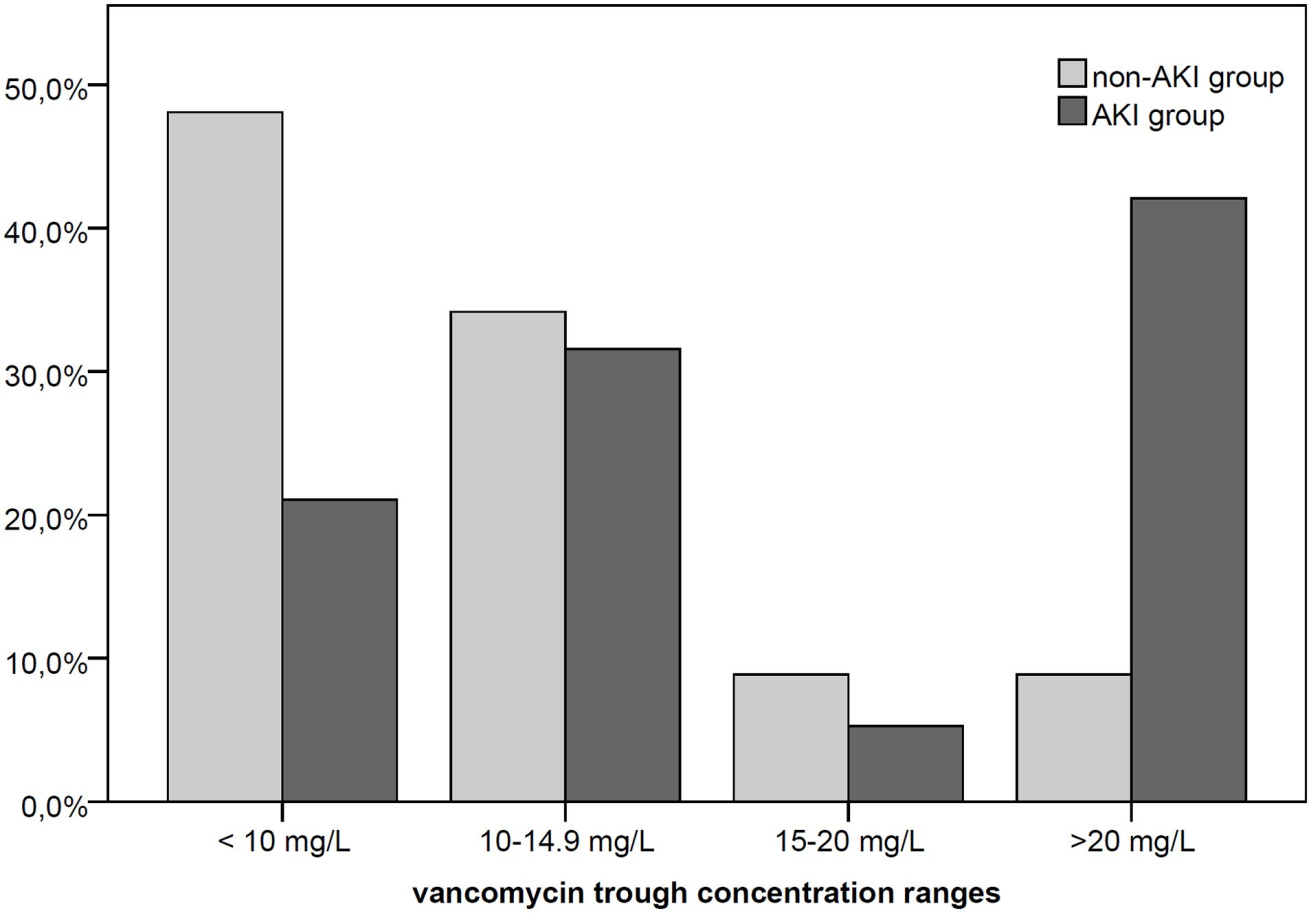
Comparative analysis of initial vancomycin trough levels according development of AKI. AKI, acute kidney injury.

The group of patients with AKI (AKI group) showed higher trough levels than patients that did not develop AKI (non-AKI group): 14.85 mg/L (IQR 11.21–21.97) vs 10.22 mg/L (IQR 7.19–13.46), p = 0.001. The proportion of patients with initial trough vancomycin concentrations >20 mg/L was significantly higher in AKI group (8; 42.1%) than the proportion encountered in the non-AKI group (7; 8.9%) (p = 0.006). On the other hand, only 20% of those with subtherapeutic levels presented AKI (Fig 2).

### Development of AKI and associated risk factors

Regarding the incidence of AKI, nineteen patients (19.4%) developed kidney dysfunction, mostly KDIGO stage 1 (52.6%). Among those who developed AKI, only one needed hemodialysis and six died (31.6%). For those with VAN, median time from starting vancomycin therapy until development of AKI was six days (IQR 4.0–9.0). AKI-group had higher frequency of concomitant use of piperacillin-tazobactam (47.4% vs. 8.9%, p<0.001), used higher number of nephrotoxic drugs (3 vs. 2, p = 0.031), had lower eGFR (108.5 vs 122.1, p = 0.028) and higher values of vancomycin trough levels (14.85 vs. 10.22mg/dL, p = 0.001). In the multivariate analysis, only vancomycin trough concentration (odds ratio [OR] 4.215; confidence interval [CI] 95% 1.575–11.278, p = 0.004), male sex (OR 2.900; CI 95% 1.280–6.530; p = 0.011) and concomitant use of piperacillin-tazobactam (OR 4.661; CI 95% 2.265–9.589; p <0.001) were independently associated with AKI development (Table 3). The final model of the multivariate analysis fitted well the data (Hosmer-Lemeshow test = 0.792). Overall performance was moderate (Nagelkerke R^2^ = 0.446).

**Table 3:** Model of regression of Poisson (with robust covariance matrix) for the factors related with acute kidney injury in non-critically patients receiving vancomycin.

Variables	OR (95% CI)	*p* value	Final model	p-value
Age	1.004 (0.952–1.059)	0.880	-	
Male sex	1.172 (1.013–1.356)	0.033	2.900 (1.280–6.530)	0.011
Charlson comorbidity index	1.258 (0.913–1.733)	0.160	-	
Hospital-acquired infection (or type of infection)	0.174 (0.019–1.592)	0.122	-	
Infection site	0.560 (0.002–186.366)	0.836	-	
Baseline SCr	1.385 (0.071–26.889)	0.830	-	
Baseline urea	1.004 (0.954–1.057)	0.878	-	
Number of potential nephrotoxic drugs	1.498 (0.862–2.601)	0.152	-	
Concomitant piperacillin-tazobactam	1.616 (1.248–2.092)	<0.001	4.661 (2.265–9.589)	<0.001
Concomitant polymyxin B	0.282 (0.040–2.004)	0.206	-	
Duration of vancomycin use	1.118 (0.963–1.298)	0.141	-	
Initial trough vancomycin concentration				
<10.0	1		1	
10.0–14.9	2.622 (0.504–13.647)	0.252	1.848 (0.636–5.370)	0.259
15.0–20.0	2.897 (0.205–40.985)	0.431	1.897 (0.326–11.041)	0.476
>20.0	15.255 (2.485–93.645)	0.003	4.215 (1.575–11.278)	0.004
Renal replacement therapy	2.257 (2.088–2.439)	<0.001	-	

OR, odds ratio; CI, confidence interval; SCr, serum creatinine.

## Discussion

In this study, we found that one in five non-critically ill patients using vancomycin admitted to a Brazilian public hospital developed AKI, and that supratherapeutic serum levels of vancomycin, male sex and concomitant use of piperacillin-tazobactam were independently associated with this outcome. Recent studies have reported a wide range in the incidence of VAN [4,18,19]. However, the comparison of studies was difficult due to the varied diagnostic criteria used to identify AKI. Moreover, setting and baseline characteristics of the population also differed considerably among them. In the ICU setting, Qian et al. have reported an incidence of 5.9% of AKI in a predominantly male Chinese population [4]. In another large retrospective study including 1430 critically ill patients, the frequency of AKI was 21% [19]. Finally, in a systematic review, Van Hal et al. reported incidence ratios of AKI varying from five to 43%, including several settings, such as ICU and wards [8].

Data addressing AKI in non-critically ill population using vancomycin are limited. We hypothesized that the incidence of AKI in this population would be lower than that observed in the ICU patients, because of the presumed lesser degree of disease severity. However, we found herein a high incidence of AKI, which was at least similar to that observed in studies with critically ill patients. Bosso et al. also reported similar rates of nephrotoxicity in patients using vancomycin (55/288; 19%) in a prospective multicenter study including non-critically ill patients admitted to seven hospitals in USA [20]. Another prospective multicenter study reported a lower rate of nephrotoxicity (36/370; 9.7%) [21].

Our results suggest that in a large proportion of patients, usual vancomycin dose regimen (1g every 12 hours) did not reach target vancomycin trough serum concentration, 15-20mg/L, as recommended by the Infectious Disease Society of America (IDSA) to the treatment of severe infections [11]. Studying critically ill patients, Qian et al. also reported low rates of concordance with trough vancomycin levels recommended [4]. The authors found that fixed dose regimen was not a good strategy, since it did not allow obtaining adequate vancomycin trough serum concentrations, more commonly resulting in subtherapeutic levels (58.1%). Bakke et al. performed a prospective observational study with 83 patients in Norway, and found that less than 40% of the patients achieved therapeutic trough serum concentrations during the first three days of vancomycin therapy [12]. Similar results were observed by Obara et al. in 83 Brazilian critically-ill patients [13]. Finally, another retrospective study also including 164 critically-ill patients, conducted by Tuon et al. in two Brazilian hospitals have reported that only 13.4% of patients achieved target levels [22]. Considering that most previous studies have been focused on critically ill patients, we believe our study contributes to expand the knowledge on vancomycin use in non-critically ill patients. In this context, the low level of compliance with international guidelines on treatment of infectious disease is worrisome and deserves more investigation. One strategy that could be implemented is the participation of pharmacists in dose adjustments using vancomycin serum levels. A recent study suggested that their participation in the multidisciplinary team can be relevant to improve outcomes [23].

Our data showed that VAN was independently associated with trough serum concentration >20mg/L (p = 0.004), male sex (p = 0.011) and concomitant use of piperacillin-tazobactam (p<0.001). Avoiding vancomycin levels >20mg/L is a known recommendation given by renowned societies [11,24,25]. An interesting finding was that male sex was associated with VAN. In a recent systematic review involving preclinical studies, it was reported that female sex was associated with greater toxicity of vancomycin [26]. However, our results did not corroborate this review. Therefore, further studies should be conducted to evaluate the influence of sex on the development of AKI. One hypothesis would be that differences in clinical parameters could explain, at least in part, the greater vulnerability found in men when compared to women. Concomitant use of vancomycin and piperacillin-tazobactam has been reported to have increased the risk for AKI when compared with other broad-spectrum antibiotic combinations [27]. Our findings are in line with previous results reinforcing the need for close monitoring in patients with indication of concomitant use of these drugs [18,28–30]. The mechanisms behind this finding remain to be investigated. One hypothesis is that the use of piperacillin-tazobactam may reduce clearance of vancomycin resulting in its accumulation with consequent increase in serum trough levels [18].

Among the strengths of our study, we could state that this is one of few studies prospectively designed to assess nephrotoxicity in the context of non-critically ill patients on vancomycin. In addition, it brought clearer evidence of a causal relationship between vancomycin use and the development of nephrotoxicity. However, our study presents limitations to be addressed. We included a small sample of patients in a single center design, which limits the generalizability of the results. Although our patients were not from ICU, our hospital is a reference for the assistance of complex and severe diseases, with a population of patients presenting predominantly multiple comorbidities and originally from all over the state of Minas Gerais. Higher body mass index has been associated with dosing regimen and elevated vancomycin trough levels [31]. Unfortunately, we could not obtain this variable in this study due to the lack of data on height in the charts. Another limitation was that dehydration and post-renal AKI have not been systematically evaluated in our study, even though other potential causes of renal injury, besides vancomycin toxicity, have been investigated by the assistant physicians in all patients who presented AKI. These aspects might have influenced our results. Another weakness is the lack of a control group with similar baseline characteristics that had used other glycopeptide antibiotic rather than vancomycin. Further studies should be performed to investigate pharmacokinetics parameters in a real-world scenery of patient care to bring contributions to dosing regimens of vancomycin.

In summary, the incidence of VAN can be quite elevated even in a population of non-critically ill patients. Male sex, concomitant use of piperacillin-tazobactam and vancomycin trough levels >20mg/L are independently associated with this outcome. Close monitoring of renal function and individual strategies of vancomycin dose regimen should be established. Therapeutic drug monitoring and elaboration of local protocols of vancomycin use, including the participation of pharmacists in dose adjustments are essential to improve medication safety. Additional multicenter studies with larger samples are needed to confirm our findings.

## Supporting information

S1 Table List of potentially nephrotoxic drugs. (DOCX)
