# Supplementary material for: Vancomycin-associated nephrotoxicity in non-critically ill patients admitted in a Brazilian public hospital: A prospective cohort study
Source: PLoS One. 2019 Sep 5;14(9):e0222095. doi: 10.1371/journal.pone.0222095 (PMC6728013; doi:10.1371/journal.pone.0222095)
Supplement: S1 Table — (DOCX) [file pone.0222095.s001.docx]

| Acetylsalicylic acid |
| --- |
| Acyclovir |
| Allopurinol |
| Amikacin |
| Amoxicillin- clavulanic acid |
| Amphotericin B |
| Captopril |
| Cefepime |
| Ceftriaxone |
| Cimetidine |
| Ciprofloxacin |
| Cisplatin |
| Colistin (polymyxin E) |
| Cyclosporine |
| Enalapril |
| Everolimus |
| Furosemide |
| Ganciclovir |
| Gentamycin |
| Immunoglobulin intravenous |
| Indinavir |
| Ketoprofen |
| Lansoprazole |
| Losartan |
| Manitol |
| Omeprazole |
| Penicillin potassium |
| Piperacillin-tazobactam |
| Polymyxin B |
| Rifampicin |
| Sirolimus |
| Sulfamethoxazole-thrimethoprim |
| Tacrolimus |
| Tenofovir |
| Vasopressin |
